# CenpH regulates meiotic G2/M transition by modulating the APC/C^Cdh1^-cyclin B1 pathway in oocytes

**DOI:** 10.1242/dev.141135

**Published:** 2017-01-15

**Authors:** Teng Zhang, Yang Zhou, Li Li, Zhen-Bo Wang, Wei Shen, Heide Schatten, Qing-Yuan Sun

**Affiliations:** 1 State Key Laboratory of Stem Cell and Reproductive Biology, Institute of Zoology, Chinese Academy of Sciences, Beijing 100101, China; 2 Institute of Reproductive Sciences, College of Animal Science and Technology, Qingdao Agricultural University, Qingdao 266109, China; 3 University of the Chinese Academy of Sciences, Beijing 100049, China; 4 Department of Reproductive Medicine, Guangdong Women and Children Hospital, Guangzhou 510010, China; 5 Department of Veterinary Pathobiology, University of Missouri, Columbia, MO 65211, USA

**Keywords:** CenpH, G2/M transition, Cyclin B1, APC/C^Cdh1^, Fzr1, Meiosis, Oocyte

## Abstract

Meiotic resumption (G2/M transition) and progression through meiosis I (MI) are two key stages for producing fertilization-competent eggs. Here, we report that CenpH, a component of the kinetochore inner plate, is responsible for G2/M transition in meiotic mouse oocytes. Depletion of CenpH by morpholino injection decreased cyclin B1 levels, resulting in attenuation of maturation-promoting factor (MPF) activation, and severely compromised meiotic resumption. CenpH protects cyclin B1 from destruction by competing with the action of APC/C^Cdh1^. Impaired G2/M transition after CenpH depletion could be rescued by expression of exogenous cyclin B1. Unexpectedly, blocking CenpH did not affect spindle organization and meiotic cell cycle progression after germinal vesicle breakdown. Our findings reveal a novel role of CenpH in regulating meiotic G2/M transition by acting via the APC/C^Cdh1^-cyclin B1 pathway.

## Abstract (precis)

**Summary:** CenpH, a component of the kinetochore inner plate protein, is necessary for cyclin B1 stabilization and is responsible for the G2/M transition in meiotic mouse oocytes.

## INTRODUCTION

Fully grown mammalian oocytes remain arrested at prophase I within antral follicles before gonadotropin signals induce the resumption of meiosis I (MI) and progression to meiosis II (MII). Prophase I arrest, also termed the germinal vesicle (GV) stage, is the result of low maturation-promoting factor (MPF) activity (Adhikari et al., 2012; Jones, 2004). The resumption of meiosis, referred to as germinal vesicle breakdown (GVBD), is similar to the G2/M transition of mitosis in being associated with the activation of MPF (Adhikari et al., 2012; Doree and Hunt, 2002). MPF is a complex of the catalytic subunit cyclin-dependent kinase 1 (Cdk1; also known as Cdc2) and the regulatory subunit cyclin B1 (Doree and Hunt, 2002). Activation of Cdk1 requires the association with cyclins as well as the dephosphorylation of Thr14 and Tyr15 residues by Myt1 and Wee1 protein kinases (Morgan, 1995; Nurse, 1990). Depletion of Myt1 causes partial meiotic resumption in mouse oocytes (Oh et al., 2010). Wee1B (Wee2), a key Cdk1 inhibitory kinase, is located in the GV-stage oocytes and depletion of its expression enhances meiotic resumption. Wee1B inhibits Cdk1 phosphatase activity, whereas the cell division cycle 25B (Cdc25B) phosphatase releases Cdk1 activity by dephosphorylating Wee1B-phosphorylated Cdk1 (Branzei and Foiani, 2008; Oh et al., 2010). Knockdown of Cdc25B in GV stage oocytes inhibits meiotic resumption and causes low activity of MPF (Lincoln et al., 2002). Cyclin B1 is continuously degraded by the anaphase-promoting complex/cyclosome (APC/C) during prophase I arrest (Reis et al., 2006). In GV stage mouse oocytes, Cdh1 (also called Fzr1) is required for APC/C-mediated cyclin B1 destruction to arrest at prophase I (Holt et al., 2011; Reis et al., 2006). Early mitotic inhibitor 1 (Emi1; also known as Fbxo5), an inhibitor of APC/C^Cdh1^, is responsible for cyclin B1 destruction and inactivation of MPF. Reduction of Emi1 can delay resumption of meiosis by preventing the accumulation of cyclin B1, whereas Emi1 overexpression leads to GVBD (Marangos et al., 2007). Interestingly, BubR1 (a spindle assembly checkpoint protein; also known as Bub1b) has been shown to affect prophase I arrest in mouse oocytes. BubR1-depleted oocytes spontaneously undergo GVBD in the presence of the chemical inhibitor IBMX. BubR1 knockdown can decrease the expression level of Cdh1. However, the GVBD rate in BubR1-depleted oocytes is reduced by injecting *Cdh1* cRNA into the oocyte (Homer et al., 2009). Significantly, a subunit of the NDC80 complex, Hec1 (which itself is also known as Ndc80), has also been shown to regulate meiosis resumption in mouse oocytes. Depletion of Hec1 in mouse oocytes severely affects the G2/M transition because of impaired activation of Cdk1. Unexpectedly, impaired meiosis resumption is due to instability of cyclin B2, because Hec1 can protect cyclin B2 from APC/C^Cdh1^-mediated destruction (Gui and Homer, 2013).

It is widely known that CenpH localizes to kinetochores in mammals (Alonso et al., 2007; Sugata et al., 2000, 1999). The kinetochore plays a fundamental role in accurate chromosome segregation in eukaryotes. It is a multi-protein structure that associates with centromeric DNA and binds spindle microtubules to the chromosomes, which is required for chromosome movement (Cleveland et al., 2003; Fukagawa, 2004). In particular, the centromere-specific histone H3 variant CenpA forms the platform for kinetochore assembly. Several additional components of the constitutive centromere-associated network, including CenpC, CenpH, CenpI, and CenpK through to CenpU, have been identified to associate with CenpA (Foltz et al., 2006; Fukagawa, 2004). In vertebrates, a subgroup of proteins, including CenpH, CenpI and CenpK, play essential roles in kinetochore structure and function. The CenpH and CenpI complex is a direct regulator of kinetochore-microtubule dynamics and is required for faithful chromosome segregation, and as a marker directing CenpA deposition to centromeres (Amaro et al., 2010; Cheeseman et al., 2008; Okada et al., 2006). Absence of CenpH causes severe mitotic phenotypes, including misaligned chromosomes and multipolar spindles in human cells (Orthaus et al., 2006). Indeed, CenpH also has at least one other function involving modulation of the cell cycle through an interaction with CenpC (Fukagawa et al., 2001). Interestingly, however, it is not yet known whether CenpH has other relevant roles beyond the binding of spindle microtubules to chromosomes or the chromosome segregation machinery.

Here, we investigated the role of CenpH protein in regulating the meiotic cell cycle in mouse oocytes. Unexpectedly, we show that depletion of CenpH inhibits G2/M transition by continuous degradation of cyclin B1, while the prophase I arrest induced by CenpH knockdown can be rescued by injecting exogenous cyclin B1 mRNA. Finally, we show that CenpH-dependent effects on meiotic resumption require the presence of Cdh1, thereby demonstrating that CenpH-dependent regulation of APC/C^Cdh1^ is essential for regulating prophase I arrest.

## RESULTS

### Expression and subcellular localization of CenpH during oocyte meiotic maturation

**Fig. 1.**
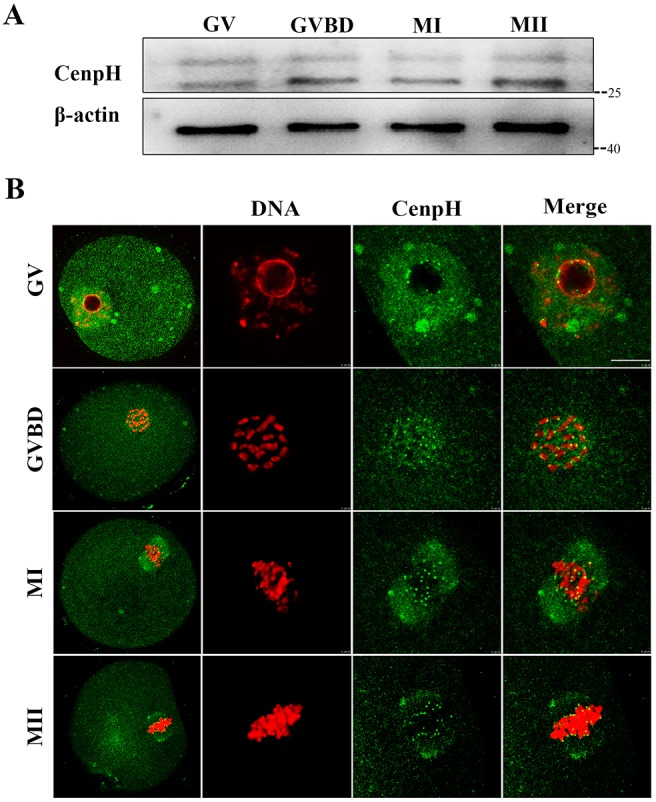
**Expression and subcellular localization of CenpH during mouse oocyte meiotic maturation.** (A) Expression of CenpH protein as revealed by western blot analysis. Samples of 200 oocytes were collected after culture for 0, 4, 8 and 12 h, representing the time points when most oocytes had reached the GV, GVBD, MI and MII stages, respectively. Marker kDa values are given to the right. (B) Confocal microscopy showing the subcellular localization of CenpH (green) in mouse oocytes at GV, GVBD, MI and MII stages. Note the localization of CenpH to kinetochores as well as to spindle and poles at MI and MII stages. Also note that nonspecific CenpH antibody binding occasionally produces punctuate staining artifacts. DNA (red) was counterstained with Hoechst 33342. Scale bar: 10 μm.

To investigate the role of CenpH during meiosis, its expression and subcellular localization were examined. Oocytes were collected after having been cultured for 0, 4, 8 or 12 h, corresponding to GV, GVBD, MI and MII stages, respectively. Immunoblotting analysis showed that CenpH protein was expressed from GV to MII stages (Fig. 1A). CenpH was more concentrated in the germinal vesicle at the GV stage (Fig. 1B). Shortly after GVBD, clear staining was observed at the kinetochores. When oocytes reached the MI and MII stages, CenpH signal was still obvious at the kinetochores of chromosomes. Subcellular CenpH localization during oocyte meiosis was similar to that in mitosis, suggesting that it might contribute to kinetochore-microtubule attachment in meiosis.

### Depletion of CenpH impairs GVBD and Cdk1 activity dependent on cyclin B1

**Fig. 2.**
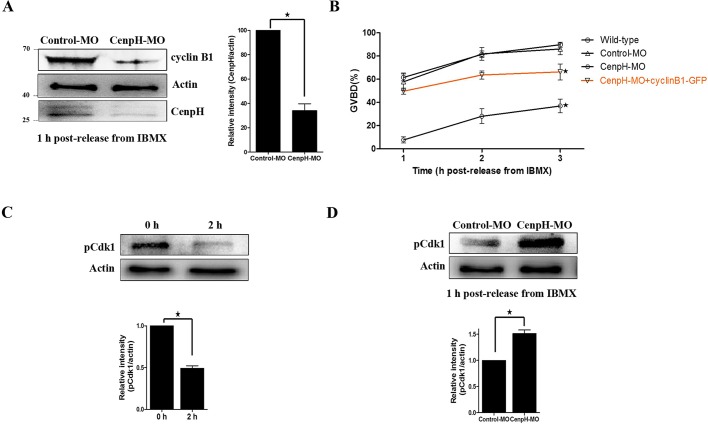
**Depletion of CenpH impairs GVBD and MPF activity.** (A) Western blotting of CenpH, cyclin B1 and β-actin in CenpH MO-injected and control MO-injected oocytes 1 h following release from IBMX (150 oocytes per sample). CenpH is 28 kDa, β-actin is 43 kDa and cyclin B1 is 55 kDa. The relative staining intensity of CenpH was assessed by densitometry. (B) GVBD rates at 1, 2 and 3 h following release from IBMX for wild-type, control MO-injected, CenpH MO injected, and CenpH MO+cyclin B1-GFP oocytes. (C) The phosphorylation level of Tyr15 of Cdk1 (pCdk1) in normal GV and GVBD oocytes. The relative staining intensity of pCdk1 was assessed by densitometry. (D) pCdk1 levels in control MO-injected and CenpH MO-injected oocytes 1 h following release from IBMX (150 oocytes per sample). The relative staining intensity of pCdk1 was assessed by densitometry. Data are mean±s.e.m. *P<0.05.

For depleting CenpH in mouse oocytes, we used a morpholino (MO) antisense microinjection approach. CenpH MO was microinjected into GV stage oocytes followed by a 24 h incubation in IBMX to deplete the protein. We found that CenpH protein was knocked down by 60-70% by MO injection compared with the control group (Fig. 2A).

Unexpectedly, only ∼37% of CenpH-depleted oocytes underwent GVBD by 3 h following release from IBMX. The percentage of oocytes at GVBD stage was significantly lower in the CenpH knockdown group than in control or wild-type oocytes (36.96±13.32% versus 86.15±11.09% or 89.82±4.75%; *P*<0.05; Fig. 2B). This indicated that CenpH-depleted oocytes had a reduced capacity for meiotic resumption. Furthermore, we examined Cdk1 activity by determining its Tyr15 phosphorylation state. We found that Cdk1 activity in CenpH-depleted oocytes was less than that of control oocytes by 1 h following release from IBMX (Fig. 2C,D). In mouse oocytes, cyclin B1 is indispensable for Cdk1 activation during the G2/M transition. We therefore examined the cyclin B1 level in CenpH-depleted oocytes and found that it was reduced (Fig. 2A). This suggested that the prophase I arrest following CenpH depletion was due to reduced cyclin B1 levels and the activity of MPF.

### Impaired GVBD after CenpH depletion can be rescued by cyclin B1 overexpression

We next examined whether exogenous cyclin B1 expression could rescue the G2/M transition defect. Overexpression of cyclin B1-GFP in CenpH-depleted oocytes increased GVBD dramatically after injecting exogenous cyclin B1 mRNA (Fig. 2B, Fig. S1E). These data suggested that CenpH is required to stabilize cyclin B1, which in turn plays an indispensable role in the G2/M transition.

### Loss of CenpH causes decreased cyclin B1 levels owing to APC/C^Cdh1^-mediated destruction

**Fig. 3.**
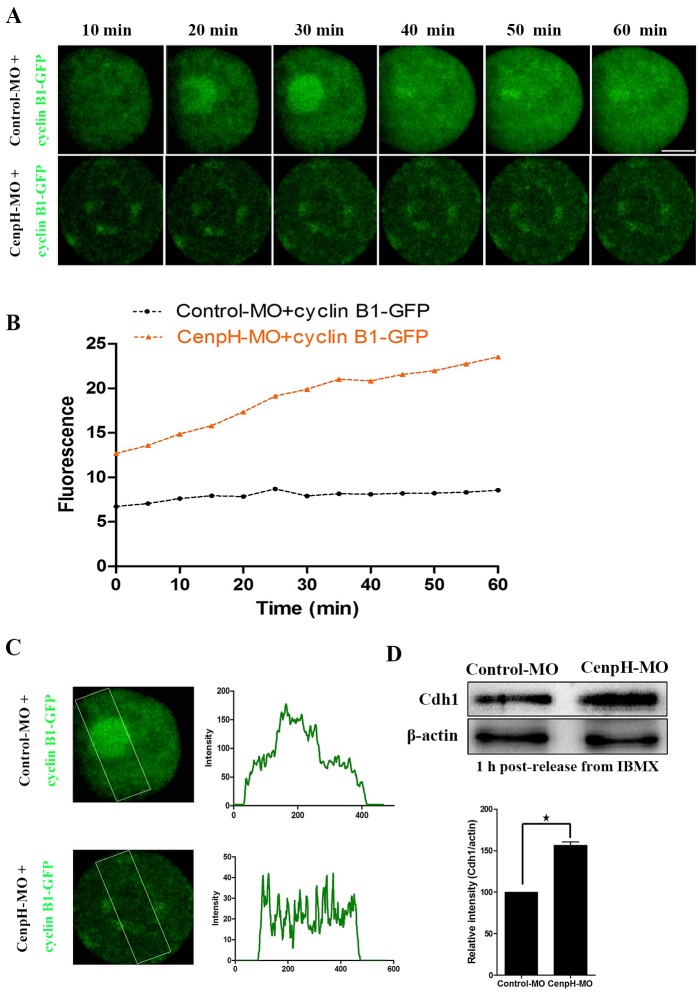
**Depletion of CenpH causes a decreased level and failed nuclear entry of cyclin B1, as well as an increased level of Cdh1 protein.** (A) CenpH MO or control MO was microinjected into GV stage oocytes followed by 20 h incubation in 200 μM IBMX. Then, cyclin B1-GFP mRNA was injected and the oocytes were maintained for 15 min in 200 μM IBMX. Live cell imaging shows the dynamics of cyclin B1-GFP at 10 min intervals. (B) The relative fluorescence intensity of cyclin B1-GFP assessed by densitometry. (C) The accumulation pattern of cyclin B1-GFP in the nucleus of CenpH MO-injected or control MO-injected oocytes. The boxed area was assessed for fluorescence intensity of each oocyte. (D) Western blotting of Cdh1 and β-actin in the CenpH MO-injected and control MO-injected oocytes 1 h following release from IBMX (150 oocytes per sample). Relative staining intensity of Cdh1 was assessed by densitometry. Data are mean±s.e.m. *P<0.05. Scale bar: 20 μm.

To further confirm that CenpH depletion causes decreased levels of cyclin B1, we monitored the dynamics of cyclin B1-GFP accumulation after microinjection of exogenous cyclin B1-GFP mRNA in GV stage oocytes injected with control or CenpH MO. Loss of CenpH caused a 50% decrease in cyclin B1-GFP accumulation compared with controls (Fig. 3A,B). Decreased cyclin B1-GFP accumulation in CenpH-depleted oocytes indicates that the effects of CenpH depletion are caused by cyclin B1 destabilization rather than defects in translation. Cyclin B1 translocation into the nucleus is a prerequisite for MPF activation and the occurrence of GVBD. Live cell imaging following injection of exogenous cyclin B1-GFP mRNA demonstrated the ability of the cyclin B1-GFP to accumulate in the nucleus. By contrast, in CenpH-depleted oocytes, cyclin B1-GFP failed to accumulate in the nucleus (Fig. 3A,C).

In mouse oocytes, APC/C^Cdh1^-mediated destruction of cyclin B1 is indispensable for prophase I arrest. Consequently, alterations in Cdh1 levels characterize the conditions that perturb cyclin B1 accumulation and meiotic resumption. When we examined Cdh1 in CenpH-depleted oocytes using an antibody we found that levels were increased (Fig. 3D). This suggested that the prophase I arrest following CenpH depletion was due to increased APC/C^Cdh1^ activity.

### CenpH is not necessary for spindle formation and progression through MI

CenpH, as a component of the kinetochore inner plate, contributes to kinetochore-microtubule attachment in mitosis. As mentioned above, ∼37% of CenpH MO-injected oocytes underwent GVBD by 3 h following release from IBMX. These oocytes were used for subsequent observations. These MO-injected CenpH-depleted oocytes showed normal spindle assembly, chromosome alignment and polar body extrusion (Fig. S1A,B). As this might be caused by insufficient knockdown of CenpH protein, we further examined the effect of blocking CenpH on MI spindle formation and meiotic cell progression after GVBD.

**Fig. 4.**
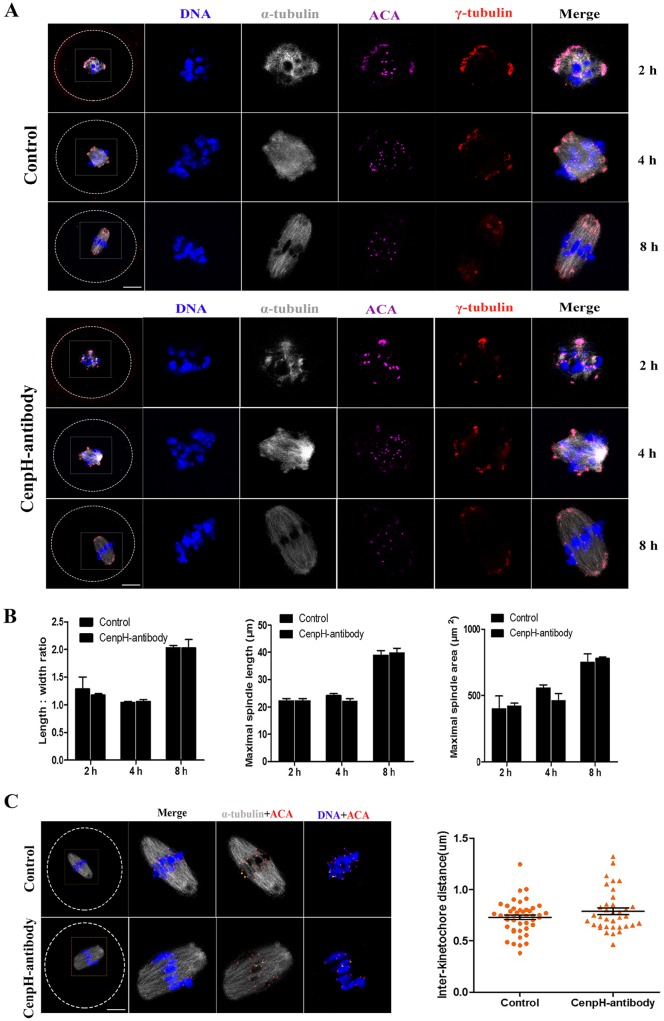
**CenpH is not required for spindle assembly.** CenpH antibody was microinjected into GVBD stage oocytes. (A) Confocal images of control and CenpH antibody-injected oocytes immunostained for DNA, kinetochores (ACA), microtubule organizing center (γ-tubulin) and microtubules (α-tubulin). (B) Length:width ratios of spindles, maximal spindle lengths and spindle areas for control and CenpH antibody-injected oocytes at 2 h (*n*=20 and *n*=12), 4 h (*n*=20 and *n*=14) and 8 h (*n*=18 and *n*=14). (C) Confocal images of control and CenpH antibody-injected oocytes stained for DNA and immunostained for kinetochores (ACA) and microtubules (α-tubulin) at 8 h (*n*=18 and *n*=14). The interkinetochore distance of control and CenpH antibody-injected oocytes was assessed. Data are mean± s.e.m. The total numbers of analyzed oocytes are indicated (*n*). Scale bars: 20 μm.

**Fig. 5.**
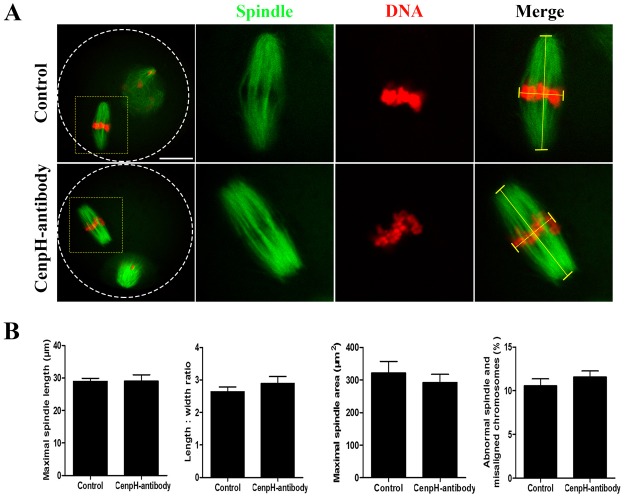
**CenpH is not necessary for MII spindle morphology.** CenpH antibody was microinjected into GVBD stage oocytes. (A) Confocal images of control and CenpH antibody-injected oocytes stained for DNA and immunostained for microtubules (α-tubulin) at 14 h. (B) Maximal spindle lengths, length:width ratios and spindle areas for control and CenpH antibody-injected oocytes at 14 h (*n*=20 and *n*=16). The right-hand chart shows the percentage of abnormal spindle and misaligned chromosomes in control and CenpH antibody-injected oocytes. The total numbers of analyzed oocytes are indicated (*n*). Data are mean±s.e.m. Scale bar: 20 μm.

First, CenpH antibody was microinjected into GV stage oocytes. Blocking of CenpH protein function by antibody injection caused prophase I arrest similar to that in the CenpH MO-injected oocytes (Fig. S1C). This result suggested that this antibody could be used to disrupt protein function to determine the role of CenpH in mouse oocytes. The CenpH antibody was microinjected into GVBD stage oocytes. Interestingly, oocytes injected with CenpH antibody extruded the first polar body at a rate similar to those of control oocytes (Fig. S1D). In mouse oocytes, shortly after GVBD, the earliest stage of spindle assembly is characterized by a spherical spindle with clumped chromosomes. Subsequently, at 4-8 h, the spindle becomes molded into a barrel-shaped bipolar structure with the chromosomes aligned on the metaphase plate (Fig. 4A). Strikingly, after blocking CenpH, we found that indexes of spindles were not significantly affected after oocyte GVBD (Fig. 4B). In addition, there was no significant difference in interkinetochore distance, as revealed by anti-centromere antigen (ACA) immunostaining, between control and CenpH antibody-injected oocytes (Fig. 4C). We also examined MII spindle morphology and found that indexes of MII spindles were not significantly affected in CenpH antibody-injected compared with control oocytes (Fig. 5A,B). Overall, these data show that, unlike in mitosis, CenpH does not play key roles in spindle formation and the MI/MII transition during mouse oocyte meiosis.

## DISCUSSION

CenpH is an important member of the family of kinetochore proteins that play a crucial role in regulating the mitotic cell cycle (Matson et al., 2012; Orthaus et al., 2006; Zhu et al., 2015). Here, we provide the first definitive evidence that CenpH has a role in regulating the meiotic G2/M transition via APC/C^Cdh1^-cyclin B1 in oocytes, differing distinctly from the effects observed in mitosis.

In mouse oocytes, CenpH was expressed throughout meiotic maturation (Fig. 1A). Inherently, the subcellular localization of CenpH is not very different from that of mitosis: the signal was still obvious at the kinetochores at meiotic metaphase (Fig. 1B). Furthermore, at the GV stage, it is more concentrated in the germinal vesicle, suggesting that CenpH might play an important role at this stage. We knocked down CenpH in GV stage oocytes by MO microinjection. Unexpectedly, ∼63% of CenpH-depleted oocytes still maintain prophase I arrest by 3 h following release from IBMX (Fig. 2B). This indicates that the expression of CenpH at the GV stage is essential for oocyte meiotic resumption. The mouse oocyte provides an ideal model for studying G2/M transition, since the oocyte is arrested at the prophase of MI for an extensive period before being released from the inhibitory microenvironment. It is well known that MPF is a key regulator of G2/M transition in mouse oocytes (Jones, 2004). The Cdk1-activating cyclin at the boundary of G2/M in mouse oocytes is cyclin B1, the APC/C^Cdh1^-mediated destruction of which is indispensable for preventing MPF activation during G2 arrest (Marangos and Carroll, 2008; Reis et al., 2006). Consequently, alterations in cyclin B1 and/or Cdh1 levels characterize many conditions that perturb Cdk1 activity and meiotic resumption (Holt et al., 2011; Marangos and Carroll, 2008; Reis et al., 2006). Our results show that reduction in cyclin B1 levels following CenpH depletion is due to increased APC/C^Cdh1^activity (Fig. 2A, Fig. 3D), indicating that the CenpH-dependent inhibition of APC/C^Cdh1^ in mouse oocytes is important for the control of G2/M transition. Thus, G2/M transition is critically poised by the competing actions of APC/C^Cdh1^ and CenpH. Analogously, prophase I arrest in mouse oocytes is controlled by Emi1-dependent regulation of APC/C^Cdh1^ (Marangos et al., 2007). By contrast, in mouse oocytes, BubR1 sustains Cdh1 levels in prophase I arrest (Homer et al., 2009). The physiological regulation of APC/C^Cdh1^ by kinetochore proteins that allows for a timely meiotic resumption is a topic for future investigation.

The importance of CenpH in balancing the G2/M transition is shown by the effects of CenpH depletion on the timing of meiotic resumption. APC/C^Cdh1^ is apparently the target of CenpH in mediating meiotic resumption because CenpH depletion leads to a reduction in the APC/C^Cdh1^ substrate cyclin B1 (Fig. 2A). Live cell imaging following injection of exogenous cyclin B1-GFP showed that loss of CenpH causes a 50% decrease in the rate of cyclin B1-GFP accumulation (Fig. 3A,B), indicating that the effects of CenpH depletion are caused by cyclin B1 destabilization rather than any defect in translation. This increase in cyclin B1 instability is likely to explain the much-attenuated increase in MPF activity during the meiotic resumption progression of CenpH-depleted oocytes. Cyclin B1 accumulation in the nucleus is a prerequisite for MPF activation and meiotic resumption (Holt et al., 2010). Furthermore, APC/C^Cdh1^ protects the oocyte from precocious GVBD by preventing nuclear accumulation of cyclin B1. In CenpH-depleted oocytes, cyclin B1-GFP failed to accumulate in the nucleus and thus inhibited MPF activity, which explains the severely compromised meiotic resumption, together with an increased level of Cdh1 (Fig. 3C,D). Significantly, impaired GVBD could be restored in CenpH-depleted oocytes by co-expressing exogenous cyclin B1 mRNA (Fig. 2B). A recent observation has shown that APC/C^Cdh1^ is necessary for maintaining cyclin B1 stabilization in mouse oocytes (Reis et al., 2006). Our results show that meiotic resumption is critically poised by the competing actions of APC/C^Cdh1^ and CenpH. Thus, CenpH is required to stabilize cyclin B1, which in turn plays an indispensable role in the MPF activation required for meiotic resumption.

Our observations might have wider implications for the role of CenpH in mammalian somatic cells. CenpH has been shown to contribute to kinetochore-microtubule attachment and accurate chromosome segregation in the mitotic cell cycle (Matson et al., 2012; Orthaus et al., 2006; Zhu et al., 2015). We found that meiotic resumption is impaired by CenpH antibody injection in GV stage oocytes (Fig. S1C), indicating the availability of an antibody that inhibits CenpH protein function. However, in contrast to mitosis, we found that blocking CenpH protein function in GVBD stage oocytes by antibody injection did not influence cell cycle progression (Fig. S1D). Interestingly, early-stage spindle assembly and MII spindle morphology were not affected by inhibiting CenpH at GVBD stage (Figs 4 and 5). Similarly, upon MO injection, 37% of CenpH-depleted oocytes underwent GVBD by 3 h following release from IBMX, and these oocytes could still extrude the first polar body (Fig. S1A). Yet, it is not clear whether the CenpH level is actually reduced in these GVBD oocytes. Subsequently, CenpH MO was injected into GV stage oocytes followed by 24 h incubation in IBMX to deplete the protein. When the CenpH protein level in control and CenpH MO-injected oocytes (GV or GVBD) was assessed by western blot 2 h following release from IBMX, it was found to be significantly decreased in the GV or GVBD oocytes compared with the control (Fig. S1F). This indicates that, unlike mitosis, CenpH is not required for spindle assembly and meiotic cell cycle progression after GVBD. An alternative interpretation of these results is that pre-existing trace amounts of CenpH in GVBD oocytes are sufficient for meiotic spindle assembly and further cell cycle progression.

**Fig. 6.**
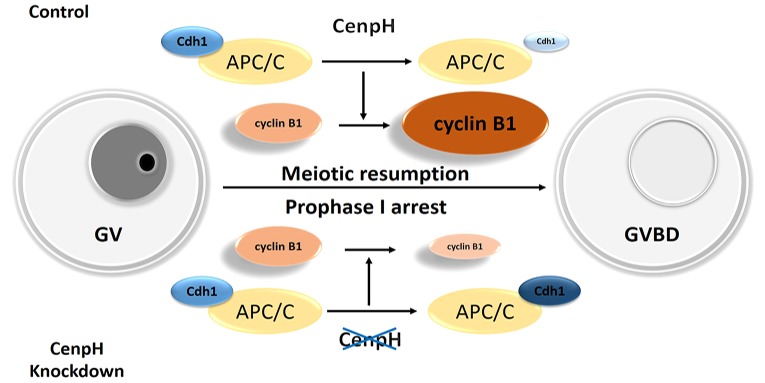
**Proposed effects of CenpH depletion on meiotic resumption in mouse oocytes.** Depletion of CenpH in mouse oocytes leads to elevated APC/C^Cdh1^ activity and instability of cyclin B1. APC/C^Cdh1^ is apparently the target of CenpH in mediating meiotic resumption. Increased expression of APC/C^Cdh1^ perturbs cyclin B1 accumulation, resulting in prophase I arrest. Consequently, CenpH is crucial in balancing the G2/M transition in mouse oocytes.

Our data uncover a major feature of CenpH in mouse oocytes that is not shared with mitosis. It is involved in APC/C^Cdh1^-mediated cyclin B1 proteolysis during prophase (Fig. 6). CenpH is necessary for cyclin B1 stabilization, which in turn plays an indispensable role in MPF activation. Moreover, APC/C^Cdh1^ is apparently the target of CenpH in mediating meiotic resumption. Thus, CenpH deficiency could have significant consequences for fertility by increasing the propensity for prophase I-arrested oocytes.

## MATERIALS AND METHODS

### Antibodies

Mouse polyclonal anti-CenpH antibody used for western blot and immunofluorescence was purchased from Abcam (ab88593); mouse monoclonal anti-α-tubulin-FITC antibody and mouse monoclonal anti-γ-tubulin antibody were obtained from Sigma-Aldrich (F2168 and T6557); human polyclonal anti-ACA antibody was purchased from Antibodies Incorporated (15-234); mouse monoclonal anti-cyclin B1 antibody was purchased from Santa Cruz (sc-245); rabbit polyclonal anti-Cdc2 (Cdk1) p34 (Tyr15) antibody was purchased from Santa Cruz (sc-12340-R); mouse polyclonal anti-β-actin antibody was purchased from Santa Cruz (sc-8432); FITC-conjugated goat anti-mouse IgG and TRITC-conjugated goat anti-mouse IgG were purchased from Zhongshan Golden Bridge Biotechnology (ZF-0312 and ZF-0313).

### Oocyte collection and culture

Care and handing of 6- to 8-week-old ICR mice was conducted in accordance with the policies of the Ethics Committee of the Institute of Zoology, Chinese Academy of Sciences. Oocytes were cultured in M16 medium (M7292, Sigma) supplemented with 200 μM 3-isobutyl-1-methylxanthine (IBMX) to maintain them at GV stage. After specific treatments, oocytes were washed thoroughly and cultured in M2 medium (M7167, Sigma) to specific stages.

### Microinjection of CenpH MO or cyclin B1-GFP mRNA

Microinjections were performed using a Narishige microinjector and completed within 30 min. For CenpH knockdown in mouse oocytes, CenpH MO (5′-ACGCCACAGAAAATAACCCAGCAGT-3′) (Gene Tools) was diluted with nuclease-free water (Sigma) to obtain a 2 mM stock. The same amount of negative control MO was used for the control. Each oocyte was microinjected with 5-10 pl of MO. After microinjection, GV oocytes were cultured for 24 h in M2 medium supplemented with 200 μM IBMX for the depletion of CenpH. For cyclin B1-GFP overexpression, 1 μg/μl cyclin B1 mRNA solution was injected into the cytoplasm of GV oocytes. For cyclin B1-GFP dynamic analysis, 20 ng/μl cyclin B1 mRNA solution was injected into the cytoplasm of the GV oocytes. For protein expression, oocytes were arrested at GV stage in M2 medium supplemented with 200 μM IBMX for 1 h. The same amount of GFP mRNA was injected as control. For blocking of CenpH protein function, GV or GVBD stage oocytes were microinjected with 5-10 pl CenpH antibody (0.5 mg/ml). After antibody microinjection, oocytes were cultured in M2 medium to specific stages.

### Immunofluorescence analysis

Immunofluorescence was performed as described (Zhang et al., 2015). For immunofluorescent staining, oocytes were fixed in 4% paraformaldehyde in PBS for 30 min at room temperature. After permeabilization with 0.5% Triton X-100 for 20 min, they were blocked in 1% BSA in PBS for 1 h at room temperature. For staining of CenpH, ACA and γ-tubulin, oocytes were incubated overnight at 4°C with anti-CenpH (1:100), anti-ACA (1:40) or anti-γ-tubulin (1:200) antibodies. After three washes in washing buffer, oocytes were incubated with FITC-conjugated goat-anti-mouse IgG (1:100) or cy5-conjugated goat anti-human IgG (1:500) or TRITC-conjugated goat anti-mouse IgG (1:100) for 2 h at room temperature. For α-tubulin staining, oocytes were incubated with anti-α-tubulin-FITC antibodies for 2 h at room temperature, oocytes were then washed three times in wash buffer and co-stained with Hoechst 33342 (10 mg/ml in PBS) for 15 min. These oocytes were mounted on glass slides and examined with an LSM 780 META confocal laser-scanning microscope (Zeiss).

### Immunoblotting analysis

Immunoblotting was performed as described (Zhang et al., 2016). Briefly, a total of 100 mouse oocytes were collected in 7 μl 2×SDS buffer and heated for 5 min at 100°C. The proteins were separated by SDS-PAGE and then transferred to PVDF membranes. Following transfer, the membranes were blocked in TBST containing 5% BSA for 2 h at room temperature, followed by incubation overnight at 4°C with mouse polyclonal anti-CenpH (1:500), mouse monoclonal anti-cyclin B1 (1:1000), rabbit polyclonal anti-Cdc2 (Cdk1) p34 (Tyr15) (1:1000) or mouse polyclonal anti-β-actin (1:1000) antibody. After three washes in TBST for 10 min each, the membranes were incubated with 1:1000 HRP-conjugated goat anti-mouse, anti-rabbit or anti-goat IgG for 1 h at 37°C. Finally, the membranes were processed using the enhanced chemiluminescence detection system (Bio-Rad). The CenpH antibody recognized two bands on the western blot: a 28 kDa band close to the expected molecular weight for CenpH; and a higher molecular weight band that might represent some form of post-translational modification.

### Time-lapse live imaging experiments

Cyclin B1-GFP dynamics were filmed on a Perkin-Elmer Ultra VIEW VOX confocal imaging system. A narrow band pass EGFP filter set and a 30% cut neutral density filter from Chroma were used. Exposure time ranged between 300 and 800 ms depending on cyclin B1-GFP fluorescence levels. The acquisition of digital time-lapse images was controlled by IP Lab (Scanalytics) or AQM6 (Andor/Kinetic imaging) software packages. Confocal images of cyclin B1 in live oocytes were acquired with a 10× oil objective on a Zeiss spinning disk confocal microscope (Perkin-Elmer).

### Image analysis

Images were acquired using the LSM 780 META confocal laser-scanning microscope equipped with a C-Apochromat 40× water-immersion objective. Data analysis was performed using ZEN 2012 LSM software (Zeiss) and ImageJ (NIH).

### Statistical analysis

Data (mean±s.e.m.) were generated from replicates that were repeated at least three times per experiment and analyzed by ANOVA using SPSS Statistics (IBM) followed by Student–Newman–Keuls test. *P*<0.05 was considered statistically significant.
